# Temperature Sensitivity of Microbial Litter Decomposition in Freshwaters: Role of Leaf Litter Quality and Environmental Characteristics

**DOI:** 10.1007/s00248-022-02041-5

**Published:** 2022-06-02

**Authors:** Silvia Monroy, Aitor Larrañaga, Aingeru Martínez, Javier Pérez, Jon Molinero, Ana Basaguren, Jesús Pozo

**Affiliations:** 1 Department of Plant Biology and Ecology, Faculty of Science and Technology, University of the Basque Country, P.O. Box 644, 48080 Bilbao, Spain; 2 Escuela de Gestión Ambiental, Pontificia Universidad Católica del Ecuador Sede Esmeraldas, Esmeraldas, 080150 Ecuador

**Keywords:** Climate change, Activation energy, Resource quality, Microbial activity, Temperature, Streams

## Abstract

Ongoing global warming is expected to alter temperature-dependent processes. Nevertheless, how co-occurring local drivers will influence temperature sensitivity of plant litter decomposition in lotic ecosystems remains uncertain. Here, we examined the temperature sensitivity of microbial-mediated decomposition, microbial respiration, fungal biomass and leaf nutrients of two plant species varying in litter quality. We also assessed whether the type of microbial community and stream water characteristics influence such responses to temperature. We incubated alder (*Alnus glutinosa*) and eucalypt (*Eucalyptus globulus*) litter discs in three streams differing in autumn–winter water temperature (range 4.6–8.9 °C). Simultaneously, in laboratory microcosms, litter discs microbially conditioned in these streams were incubated at 5, 10 and 15 °C with water from the conditioning stream and with a water control from an additional stream. Both in the field and in the laboratory, higher temperatures enhanced litter decomposition rates, except for eucalypt in the field. Leaf quality modified the response of decomposition to temperature in the field, with eucalypt leaf litter showing a lower increase, whereas it did not in the laboratory. The origin of microbial community only affected the decomposition rates in the laboratory, but it did not modify the response to temperature. Water quality only defined the phosphorus content of the leaf litter or the fungal biomass, but it did not modify the response to temperature. Our results suggest that the acceleration in decomposition by global warming will be shaped by local factors, mainly by leaf litter quality, in headwater streams.

**Supplementary Information:**

The online version contains supplementary material available at 10.1007/s00248-022-02041-5.

## Introduction

Climate change predictions suggest an increase in global mean air temperature of around 1.5–4.6 °C above pre-industrial levels by 2100 [1, 2]. In stream ecosystems, temperature is a critical physical property, and projected increases in air temperature can be translated into warmer water temperatures [3, 4]. Temperature is an important factor influencing the rate of chemical and biological reactions [5, 6], so it is expected to alter temperature-dependent ecosystem processes [7–9]. Plant litter decomposition in streams is a pivotal process in regulating carbon (C) and nutrient recycling at the global scale [10, 11]. Moreover, it is particularly sensitive to rising temperatures [12–14]. Inputs of terrestrial leaf litter are the primary source of energy and organic C in detritus-based ecosystems (e.g. forested headwater streams) [15], and their decomposition is a complex process governed by abiotic and biotic components that fuel aquatic food webs and drive nutrient recycling [16, 17]. Microbial decomposers, such as aquatic hyphomycetes, are primary actors in this process [18]. They convert organic matter into new microbial biomass and inorganic compounds and make detritus a more palatable and a better nutritional resource (microbial conditioning) for detritivores [19, 20]. Therefore, it is critical to understand how litter decomposition mediated by microbial decomposers will be altered by global warming since it will influence energy and nutrient transfer to higher trophic levels and determine the C release from streams.

In-stream litter decomposition rate is usually positively related to water temperature [21, 22] since temperature often stimulates fungal activities [23–25]. However, interactive effects of environmental drivers such as organic matter quality or water physicochemistry on sensitivity to temperature of decomposition process are not well understood [26, 27]. This knowledge gap challenges our ability to predict the magnitude of global warming effects on stream functioning. Evidence from terrestrial ecosystems suggests that the quality of resources may modulate the temperature sensitivity of decomposition and that low-quality substrates (i.e. high carbon to nutrient ratio and more structurally complex C compounds) may be more sensitive to temperature increase than high-quality ones [28–30]. This effect is explained by the higher net activation energy of the microbial enzymatic reactions required to metabolise structurally complex C substrates [28]. In freshwater ecosystems however, this issue is still under debate with studies providing mixed support [31–35]. Moreover, trophic status of streams could also influence the response of microbial activity and decomposition to temperature. For example, stimulation of the fungal activity and decomposition rates with a temperature rise has been reported in nutrient-enriched streams [24, 25]. This would be related to the microbial ability to take nutrients both from the substrate and the water column to fulfil their nutritional requirements more easily [19]. Additionally, microbial decomposers might respond differently to temperature depending on their thermal tolerance and optimal temperature [26, 36, 37] and their adaptability to new thermal conditions [38, 39]. Therefore, the temperature sensitivity of litter decomposition and its response to global warming could differ among streams with different thermal regimes because of adaptations of the local microbial communities.

In this study, we combined field and laboratory experiments to assess the environmental dependency of temperature sensitivity of microbial decomposition and associated functional variables (i.e. respiration, fungal biomass accrual, leaf nutrient content). We examined how leaf litter quality, thermal history of the microbial community and stream water chemistry influence the decomposition response to temperature. To accomplish this, we performed a decomposition experiment with leaf litter from high-quality alder (*Alnus glutinosa* (L.) Gaertner) and low-quality eucalypt (*Eucalyptus globulus* Labill) in three headwater forested streams with different thermal regimes. Autumn–winter mean water temperature ranged 4.3 °C from the coldest to the warmest stream, a value comparable to the increase predicted for 2100 [1]. Simultaneously, in the laboratory, litter which had been microbially conditioned in these streams was incubated at 5, 10 and 15 °C in water from their respective stream and in a control water from a fourth stream to assess the role of water chemistry on decomposition. We hypothesised that (1) as biological activity is temperature-dependent, increasing water temperature would enhance microbial respiration and fungal biomass and, thus, enhance nutrient immobilisation and decomposition rates; (2) low-quality litter (eucalypt) would decompose slower than high-quality litter (alder), but it would be more responsive to the temperature because of the higher sensitivity to temperature of recalcitrant C compounds (i.e. low-quality litter shows a higher activation energy than the high-quality one); (3) the response of microbial activity to temperature, and thus of litter nutrient content and decomposition, would be influenced by thermal history of microbial community (i.e. communities physiologically or taxonomically adapted to cold waters would be more responsive to temperature than those adapted to warmer ones as they display highly flexible cold-adapted enzymes that facilitate reactions at varying temperatures [40, 41]); and (4) temperature and dissolved nutrient content in the water would show a synergetic interaction, with stronger positive responses to temperature when nutrient availability is higher (mainly for low-quality litter) because of higher nutrient use efficiency by microbiota at higher temperatures [25].

## Materials and Methods

### Study Sites and Stream Water

We selected three temperate headwater streams located in northern Spain (Cantabrian range) (Table 1). These streams differed by about 4.3 °C for their autumn–winter mean water temperatures (ranging from 4.6 to 8.9 °C) (Table 1), which was recorded hourly with ACR SmartButton temperature loggers (ACR Systems Inc., Surrey, BC, Canada) from November 2013 to January 2014. All streams drain forested siliceous watersheds: La Calzada (S1) runs through *Fagus sylvatica* L. forest, while Peñaranda (S2) and Peñalar (S3) run through mixed deciduous forests dominated by *Quercus robur* L. Some upland areas of the S3 catchment are occupied by *E. globulus* plantations. All streams have *Alnus* in the adjacent riparian forests.

**Table 1:** Location and water physicochemical variables (mean ± SE) of the three study streams (*n* = 6) and the control stream (*n* = 5) during the study period (4 December 2013 to 29 January 2014). For water temperature, mean daily values (*n* = 174) and their range (in parenthesis) are shown. *SRP*, soluble reactive phosphorus. Superscripts indicate only among-stream differences (Tukey’s HSD, *p* < 0.05)

	S1	S2	S3	Control
Latitude (N)	43°07′05″	43°10′01″	43°18′33″	43°13′01″
Longitude (W)	03°25′59″	03°21′31″	03°15′36″	03°16′15″
Altitude (m asl)	960	460	100	265
Catchment area (km^2^)	1.05	3.60	1.89	8.01
Water temperature (°C)	4.6 (0.0–7.5)^a^	6.7 (4.0–10.3)^b^	8.9 (6.0–11.8)^c^	-
Discharge (L s^−1^)	105.9 ± 70.9	206.7 ± 167.3	104.1 ± 98.0	-
pH	7.53 ± 0.46	7.05 ± 0.65	7.27 ± 0.36	7.32 ± 0.48
Conductivity (µS cm^−1^)	44.0 ± 11.2^a^	79.7 ± 11.1^b^	70.2 ± 9.9^b^	103.6 ± 27.1^b^
Oxygen saturation (%)	102.9 ± 3.6	100.6 ± 2.9	106.4 ± 4.61	101.0 ± 6.29
NO_3_-N (µg N L^−1^)	284.4 ± 43.9^a^	250.6 ± 213.5^a^	632.8 ± 114.3^b^	427.3 ± 191.6^ab^
NO_2_-N (µg N L^−1^)	1.82 ± 0.58	2.37 ± 1.15	2.19 ± 0.89	2.65 ± 1.3
NH_4_-N (µg N L^−1^)	20.2 ± 7.1	14.15 ± 6.02	17.3 ± 8.58	21.39 ± 7.31
SRP (µg P L^−1^)	1.75 ± 0.24^a^	1.48 ± 0.88^a^	2.67 ± 1.23^ab^	4.16 ± 2.29^b^

During the study period (from 4th December 2013 to 29th January 2014), water physicochemistry was measured on six occasions. Each time, conductivity, pH and oxygen saturation were measured with a multiparametric sensor (WTW Multi 350i; Weilheim, Germany), and discharge was estimated from instantaneous water velocity as measured by a current meter (Martin Marten Z30, Current Meter). Water samples were collected from all streams on each sampling date. Then, in the laboratory, water samples were filtered (0.7 µm pore size glass fibre filters, Whatman GF/F) within 5 h after their collection and frozen (− 20 °C) for nutrient analyses. Nitrate concentration was determined by capillary ion electrophoresis (Agilent CE, Agilent Technologies, Waldbronn, Germany), and all other nutrients were analysed colorimetrically: nitrite by the sulphanilamide method, ammonium by the salicylate method and dissolved reactive phosphorus (SRP) by the molybdate method [42].

### Plant Species

We selected two riparian tree species in our study area that vary in leaf quality [43, 44]: the high-quality native *A. glutinosa* (i.e. high nutrient content and soft cuticle; hereafter “alder”) and the low-quality exotic *E. globulus* (i.e. low nutrient content, waxy cuticle and high content of oils, polyphenols and tannins; hereafter “eucalypt”). Eucalypt was selected as the representative species of low-quality litter because it occupies large areas worldwide outside its natural distribution range, including the Iberian Peninsula. It often replaces native deciduous riparian vegetation and leads to an alteration of the quality, quantity and phenology of basal resources entering stream [45], thus negatively affecting aquatic biota and whole stream functioning [46].

In November 2013, freshly fallen leaves of both species were collected from the forest floor in a single location (northern Spain, 43°12′50″ N, 3°16′10″ W) following natural abscission. The condition of the leaves was always good and without any part degraded or broken. Their coloration was homogeneous for each leaf. In the laboratory, leaf discs of 20 mm diameter were cut from the leaves using a cork borer and then air-dried to be used later in both field and laboratory experiments. Discs of this size were used instead of entire leaves in the experiment to homogenise the material among the different treatments as much as possible while containing the effort required compared to creating smaller discs.

### Field Experiment

Approximately 1.0 g (± 0.1 g) of air-dried leaf discs were enclosed in fine mesh bags (12 × 15 cm, 0.5 mm mesh size) and deployed in all streams (S1, S2, S3) on 4th December 2013. At each stream, a total of 40 bags (20 bags × 2 species) were tied to iron bars anchored to the streambeds in randomly chosen riffle sections. After 8 days of field incubation, 8 bags per species and stream were collected and used to estimate T0 mass values in the other leaf litter bags. During this period of 8 days, alder leaves lost an average of 27.8% mass (in ash free dry mass (AFDM); 26.8, 28.6 and 28.0% on average in S1, S2 and S3, respectively), and eucalyptus lost 23.4% (22.3, 22.1 and 25.7%, respectively). Thus, day 8 was considered the starting date (T0) for both the field and the laboratory experiments. This way, we discarded the initial phase of decomposition mediated primarily by abiotic mechanisms (e.g. leaching of soluble compounds) and allowed initial microbial conditioning [47]. In subsequent samplings, after 14, 28 and 48 days of incubation from T0, four bags per species and stream were collected and transported to the laboratory. Leaf discs were rinsed with filtered stream water (100 µm) over a 0.5-mm mesh sieve to remove sediment. For each bag, a set of four-leaf discs was punched out with a cork borer (12 mm diameter) for microbial respiration measurements, and another set of three discs was frozen at − 80 °C for later fungal biomass determination. We standardised the size of the discs to 12 mm for these microbial measures due to logistical requirements (small size of respirometer chambers). The rest of the remaining material was oven-dried (70 °C, 72 h) and weighed to determine leaf dry mass (DM). A portion of the DM samples were stored (− 20 °C) for later elemental analysis (C, N, P) and the rest was combusted (500 °C, 12 h) and reweighed to determine AFDM.

### Laboratory Experiment

As in the field experiment, microbial conditioning of leaf material used in the laboratory experiment was performed by incubating air-dried leaf discs (1.0 ± 0.1 g) in the three streams (S1, S2, S3). To this end, an additional 42 extra fine mesh bags per species and stream were tied to additional bars; these bags were collected after 8 days of field conditioning. The initial mass of these discs was estimated using a correction factor obtained from the bags removed at T0 (see above). Within a controlled temperature room (10 °C), nine 36 L tanks were set up as water baths with one tank per experimental temperature (5, 10 and 15 °C) and stream (S1, S2, S3) combination. A recirculating cooler (HL-160CA) was used to cool the 5 °C tanks, and a heater circulator (Julabo EH-17) was used to heat the 15 °C tanks. Each tank contained 28 microcosms (Fig. S1), which consisted of 350-mL glass cups. Microbial conditioned leaf discs from each bag were placed into microcosms: 14 microcosms contained alder discs and the other 14 contained eucalypt discs. For each tank and species, 4 microcosms contained 200 mL of filtered water (0.7 µm pore size glass fibre filters, Whatman GF/F) from the stream where each leaf material was conditioned (i.e. S1, S2 or S3; hereafter “stream water”), and the other 10 contained control water from an additional stream to rule out water physicochemical differences among the selected streams (hereafter “control water”) (see water parameters in Table 1). Therefore, each tank contained 10 replicates with control water and 4 replicates with stream water for each litter species (Fig. S1). All microcosms were constantly aerated by air pumps under a light:dark regime of 12:12 h. The experiment ran for 50 days with the water renewed every 4 days. Microcosms containing stream water were sampled once (after 50 days), and those containing control water were sampled on three occasions (after 6, 27 and 50 days). In the first two samplings (days 6 and 27), 3 microcosms were sampled, and the third sampling day was predicted (aiming for a loss of 50%). The sampling at day 50 had 4 replicates for all levels in the experiment. At each sampling, AFDM, C:N:P, microbial respiration and fungal biomass were determined.

### Nutrient Content in Decomposing Leaf Litter

Carbon (C) and nitrogen (N) concentrations (% DM) were determined using a CHNS/O elemental analyser (Perkin Elmer II), and phosphorus (P) concentration (% DM) was measured after acid digestion by the molybdenum blue method using a spectrophotometer [48].

### Fungal Biomass

Three previously frozen leaf discs from each sample were freeze-dried and weighed (DM; ± 0.1 mg) to later determine ergosterol concentration as a measure of mycelial biomass [49]. Lipid extraction and saponification were performed using KOH/methanol (8 g L^−1^) at 80 °C for 30 min in a shaking bath. Extracted lipids were then purified by solid-phase extraction (Oasis HLB cartridge, barrel size 3 cc, particle size 30 µm, pore size 80 Å; Waters Corp., Massachusetts, USA). Ergosterol was quantified by HPLC (Dionex DX-120, Sunnyvale, California, USA) by measuring absorbance at 282 nm. The HPLC detector was equipped with a Thermo Scientific Syncronis C18 (250 × 4 mm, 5 µm particle size) column (Thermo, Waltham, MA USA) and a Thermo Universal Uniguard holder for 4/4.6 mm ID3 + Syncronis C18 (10 × 4 mm, 5 µm particle size) drop in guard precolumn (Thermo, Waltham, MA USA), maintained at 33 °C. The mobile phase was 100% methanol, flowing at 1.4 mL min^−1^. Ergosterol was converted into mycelial biomass assuming 5.5 μg ergosterol mg^−1^ mycelial DM [50]. The results were expressed as mg fungal biomass g^−1^ leaf litter DM.

### Microbial Respiration

Microbial oxygen consumption rates were measured using a closed dissolved oxygen measurement system (Strathkelvin 928 System, North Lanarkshire, Scotland). Leaf discs were incubated in chambers with 3 ml of 100% dissolved O_2_ saturated filtered stream water (at 10 °C, 40 min). By measuring respiration at a standard temperature, we were including both the metabolic response related to the biomass of decomposers and the acclimation responses that microorganisms might have experienced. We decided to use a standard temperature to simplify the procedure, but we think it is a valid approach because (1) abrupt changes of 5 °C are not uncommon in headwater streams [44], (2) the short incubation time minimises the chances of registering a biomass changes during the assay, and (3) the biomass and the properties of the microorganism assemblage are expected to prevail over short-term acclimation responses. An extra chamber with only water from the respective stream was used as control. Oxygen consumption rates (mg O_2_ g^−1^ DM h^−1^) were determined by the difference in the oxygen concentration in the sample and the control over a 20 min interval and corrected for time and disc mass (dry mass (DM)).

### Data Analyses

Differences in water physicochemical characteristics among the streams were analysed using one-way ANOVA with stream as the factor. Pairwise multiple comparisons were performed by Tukey’s HSD test [51]. Decomposition rates were calculated assuming an exponential decay model [52], as follows: M_t_ = M_o_ × e^−kt^, where *k* is the decomposition rate, M_o_ is the initial mass at T0 (estimated from the set removed after 8 days of field incubation), M_t_ is the remaining mass at time t from T0 (%AFDM), and *t* is the incubation time in days. A *k* value was calculated for each replicate assuming an initial value of 100%. Field and laboratory data were statistically analysed independently. We tested the relationship among the measured variables and temperature using linear models. We fitted all the replicates in a single model per variable by means of linear mixed-effect models (LME) using the *lme4* package in R [53]. To consider the correlation among samples and to deal with the non-linear temporal pattern of some variables, the sampling time was included as a random factor in all analyses instead of including it as a covariate. We tried LME with random intercepts or random slopes and compared them with AIC, but we selected models with random intercepts in all cases due to lower AIC values. In the field experiment, *leaf species* (alder vs eucalypt) was included as a fixed factor. In the laboratory experiment, *leaf species*, *conditioning stream* (S1 vs S2 vs S3) and *water type* (stream water vs control water) were all included as fixed factors. *Temperature* was included in all analyses as a covariate. For the field experiment *DIN*, *SRP* and *DIN:SRP* of the water were also included in the models as covariates. All covariates were centred before fitting the models. For each response variable, initially, the models included all fixed factors and two-way interactions; three- and four-way interactions, as well as interactions between water nutrient levels, were not considered in the models to avoid overfitting. Collinearity among explanatory variables was very high in our study, which makes it difficult to estimate the coefficients accurately [54]. To solve this issue, variance inflation factors (VIF) were calculated for each source of variation included in the model, and those with VIF values higher than 5 were removed [54]. The removal was done sequentially by deleting the source of variation with the highest VIF. The model that had the collinearity removed was further reduced by removing sources of variation that were non-significant following ANOVA with degrees of freedom calculated with Satterthwaite approximation [55]. Nonetheless, the covariate *temperature* was kept in all models irrespective of its significance. We used restricted maximum likelihood (REML) to estimate the components of variance [56]. Similarly, we also built linear mixed-effect models to assess the sensitivity of the decomposition to temperature, with the metabolic theory of ecology (MTE) as a framework [5]. The MTE describes the temperature sensitivity as the slope (activation energy in eV) of the natural logarithm of biological activity (in our case litter decomposition) vs the inverse of the product of the absolute temperature (in K) and Boltzmann constant (8.617 × 10^–5^ eV K^−1^). We also normalised the *x* axis (Arrhenius term) by the standard temperature (10 °C) (1/kTc – 1kT, where Tc is the normalisation temperature and T is observed temperature). We calculated MTE slopes and 95% confidence intervals for each species and treatment to examine whether the temperature dependence (slope) varied among all study cases. Statistical significance was set in *p* < 0.05. All statistical analyses were conducted using R statistical software version 3.2.2 [57].

## Results

### Stream Water Characteristics

The mean water temperature differed significantly among the streams and exhibited a 4 °C range from the coldest to the warmest stream (Table 1) (ANOVA, *F*_2, 174_ = 195.96, *p* < 0.0001). All streams, including the control stream, presented circum-neutral pH and well oxygenated waters (Table 1). Even though SRP concentration was low in all streams; the control stream had a higher SRP value than S1 and S2 (Table 1) (ANOVA, *F*_3,19_ = 4.54,* p* = 0.01). S3 showed higher nitrate concentration than the other streams (Table 1) (ANOVA, *F*_3,19_ = 7.63,* p* = 0.002).

### Litter Decomposition Rate

**Fig. 1:**
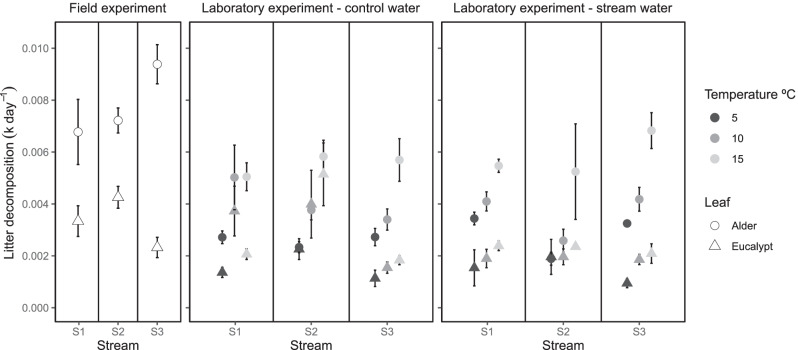
Leaf decomposition (k, day^−1^) of alder (circle) and eucalypt (triangle) litter after incubation in the field and in the laboratory (stream water vs control water at 5 °C, 10 °C and 15 °C). Note that the colour legend does not apply to field data. S1, coldest stream; S2, intermediate stream; S3, warmest stream. Mean and SE are shown

In the field experiment, the decomposition rate of alder was faster than that of eucalypt, but both species responded differently to temperature (1st and 2nd hypothesis, H1 and H2) (Fig. 1 and Fig. S2; Table 2). Alder decomposition rate increased with stream water temperature and ranged from 0.0063 day^−1^ at the coldest stream (S1) to 0.0094 day^−1^ at the warmest one (S3) (Fig. 1). By contrast, for the eucalypt litter, the lowest and the highest decomposition rate was observed at the warmest stream (S3, 0.0023 day^−1^) and at the stream with the intermediate temperature (S2, 0.0043 day^−1^), respectively (Fig. 1 and Fig. S2). In the laboratory experiment, the decomposition of eucalypt litter was also slower than that of alder and was also more variable among treatments (Fig. 1 and Fig. S2; Table 3). The decomposition rate depended on the stream of leaf conditioning (Table 3). In the laboratory, the decomposition rates of both species increased with water temperature (H1) (Fig. 1 and Fig. S2; Table 3 and Table S1), but the stream of leaf conditioning and type of water did not influence the response to temperature (3rd and 4th hypothesis, H3 and H4; Fig. 1 and Fig. S2; Table 3).

**Table 2:** Results of linear mixed-effect model testing effects of leaf litter type and stream temperature on leaf decomposition, microbial respiration, fungal biomass accrual and leaf nutrients (%N, %P), associated with alder and eucalypt leaves incubated in the field. Significant values (*p* < 0.05) are highlighted in bold

	Df	*F* value	*p* value	Coef. sign	Interpretation
Leaf decomposition					
Temperature	1.63	1.22	0.2738		
Leaf	1.63	58.48	** < 0.0001**	** − **	Alder > Euc
Temperature: leaf	1.63	5.70	**0.0200**	** − **	Temperature stimulation smaller on eucalypt
Respiration					
Temperature	1.64	0.09	0.7683		
Leaf	1.64	136.31	** < 0.0001**	** − **	Alder > Euc
Temperature: leaf	1.64	4.57	**0.0364**	** + **	Temperature inhibition smaller on eucalypt
Fungal biomass					
Temperature	1.48.9	5.56	**0.0224**	** + **	Temperature stimulates
Leaf	1.46.9	57.94	** < 0.0001**	** − **	Alder > Euc
Water SRP	1.47.6	20.71	** < 0.0001**	** − **	SRP inhibits
Water DIN:SRP	1.47.6	9.55	**0.0033**	** − **	DIN:SRP inhibits
% nitrogen					
Temperature	1.64.7	0.68	0.4141		
Leaf	1.63.2	896.76	** < 0.0001**	** − **	Alder > Euc
% phosphorus					
Temperature	1.62.3	0.10	0.7495		
Leaf	1.62.0	49.01	** < 0.0001**	** − **	Alder > Euc
Temperature: leaf	1.62.0	8.69	**0.0045**	** + **	Temperature stimulation larger on eucalypt

**Table 3:** Results of linear mixed-effect model testing effects of water temperature (5, 10, 15 °C), leaf litter type (alder, A; eucalypt, E), stream of leaf conditioning (S1, S2, S3) and water type (control or stream water) on leaf decomposition, microbial respiration, fungal biomass accrual and leaf nutrients (%N, %P) associated with alder and eucalypt leaf discs incubated in the laboratory. Significant values (*p* < 0.05) are highlighted in bold

	Df	*F* value	*p* value	Coef. sign	Interpretation
Leaf decomposition					
Temperature	1,243.0	42.62	** < 0.0001**	** + **	Temperature stimulates
Leaf	1,243.0	20.59	** < 0.0001**	** − **	A > E
Microbial community	2,243.0	9.50	**0.0001**	**S2 ( +), S3 ( −)**	S2 > S1 > S3
Respiration					
Temperature	1,209.9	31.96	** < 0.0001**	** − **	Temperature inhibits
Leaf	1,209.7	528.10	** < 0.0001**	** − **	A > E
Fungal biomass					
Temperature	1,208.9	5.23	**0.0232**	** + **	Temperature stimulates
Leaf	1,208.9	189.14	** < 0.0001**	** − **	A > E
Water quality	1,199.8	8.23	**0.0046**	** + **	Water from control stream stimulates
% nitrogen					
Temperature	1,245.2	0.18	0.6741		
Leaf	1,245.2	4221.4	** < 0.0001**	** − **	A > E
% phosphorus					
Temperature	1,243.8	0.21	0.6490		
Leaf	1,243.8	314.09	** < 0.0001**	** − **	A > E
Water quality	1,205.3	19.27	** < 0.0001**	** + **	Water from control stream stimulates

**Fig. 2:**
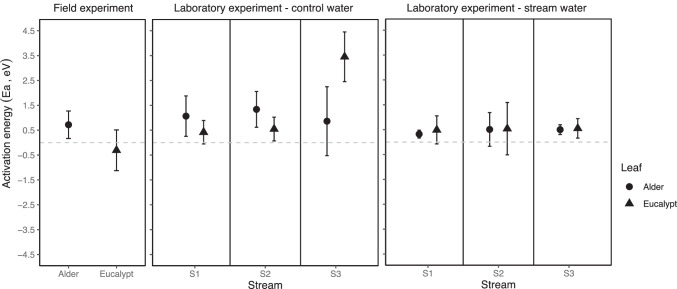
Sensitivity to temperature (activation energy, eV) of litter decomposition, for alder (circle) and eucalypt (triangle) in the field and the laboratory experiment (stream water vs control water). Streams are identified as S1 (coldest), S2 (intermediate), S3 (warmest). Mean slope ± confidence intervals (95% CI) are shown

When the sensitivity to temperature was expressed as activation energy (Ea), its value for alder decomposition in the field was 0.71 eV (Fig. 2). By contrast, Ea for eucalypt was more variable and not significantly different from 0 in most cases (95% CI =  − 1.07–0.45) (Fig. 2 and Fig. S3a; Tables S1 and S2). In the laboratory experiment, Ea for both leaf species was not influenced by stream of leaf conditioning and the type of water (H3 and H4; Fig. 2 and Fig. S3b and c; Tables S1 and S2).

### Microbial Respiration

**Fig. 3:**
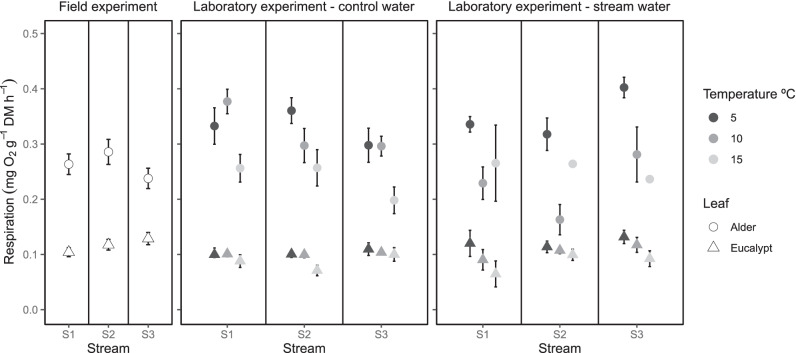
Oxygen consumption (mg O_2_ g^−1^ DM h^−1^) in alder (circle) and eucalypt (triangle) leaf discs after incubation in the field and in the laboratory (stream water vs control water at 5 °C, 10 °C and 15 °C). Please note that the colour legend does not apply to field data. S1, coldest stream; S2, intermediate stream; S3, warmest stream. Mean and SE are shown

In litter discs from the field experiment, microbial respiration rate (measured at 10 °C) was lower for eucalypt litter than for alder (Fig. 3 and Fig. S4; Table 2). Only the respiration rate of eucalypt was related to stream water temperature (H2) (Table 2), and it tended to be higher for litter incubated in the warmest stream (S3) (Fig. 3 and Fig. S4; Table 2). In the laboratory experiment, the respiration rate for eucalypt litter was also lower than that of alder (Fig. 3 and Fig. S4; Table 3). The respiration rate of both species was related to water temperature (H1) (Fig. 3 and Fig. S4; Table 3). In all treatments, leaf discs incubated in microcosms at 5 °C had higher respiration rates than discs incubated at 15 °C when the respiration was measured at standard temperature of 10 °C (Fig. 3 and Fig. S4; Table 3). The respiration rate of litter from different conditioning streams or incubated with different type of water did not influence the response of respiration to temperature (H3 and H4) (Fig. 3 and Fig S4; Table 3).

### Fungal Biomass Accrual

**Fig. 4:**
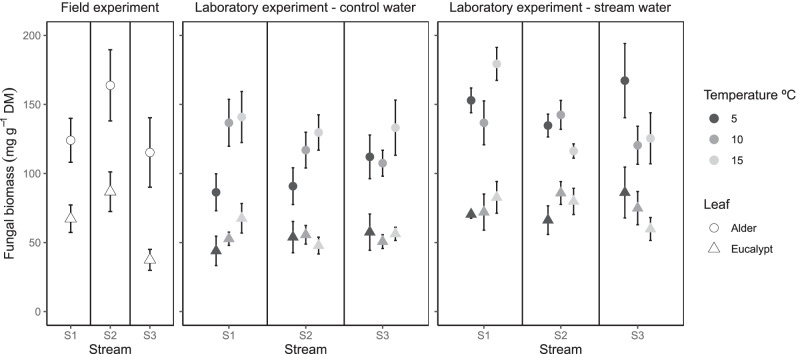
Fungal biomass (mg g^−1^ DM) in alder (circle) and eucalypt (triangle) leaf discs after incubation in the field and in the laboratory (stream water vs control water at 5 °C, 10 °C and 15 °C). Note that the colour legend does not apply to field data. S1, coldest stream; S2, intermediate stream; S3, warmest stream. Mean and SE are shown

In the field experiment, fungal biomass tended to be higher on day 28 (Fig. S5), attaining maximum values of 257 mg g^−1^ DM and 123 mg g^−1^ DM on alder and eucalypt litter, respectively, in the S2 stream (intermediate temperature) (Fig. 4 and Fig. S5; Table 2). For both species, fungal biomass was higher at higher stream water temperature (H1 and H2) (Fig. 4 and Fig. S5; Table 2). The response of fungal biomass to temperature was not influenced by stream of leaf conditioning (H3) (Fig. 4 and Fig. S5; Table 2). Fungal biomass showed a negative relationship with SRP concentration in the water and DIN:SRP, but it did not influence its response to temperature (H4) (Fig. 4 and Fig. S5; Table 2). In the laboratory experiment, fungal biomass was also higher on alder litter than on eucalypt (Fig. 4 and Fig. S5; Table 3). In the laboratory, fungal biomass on both litter species was positively related to temperature (H1). Its responses to temperature did not differ among litter from different conditioning streams (H3) or between type of water (H4) (Fig. 4 and Fig. S5; Table 3).

### Nitrogen and Phosphorus in Decomposing Leaf Litter

**Fig. 5:**
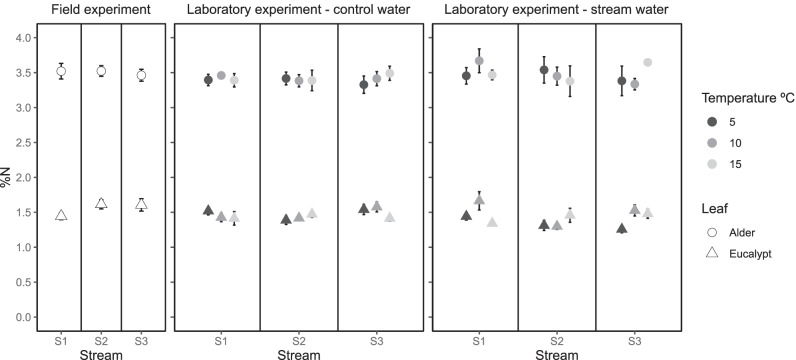
Nitrogen concentration (% DM) in alder (circle) and eucalypt (triangle) leaf discs after incubation in the field and in the laboratory (stream water vs control water at 5 °C, 10 °C and 15 °C). Note that the colour legend does not apply to field data. S1, coldest stream; S2, intermediate stream; S3, warmest stream. Mean and SE are shown

**Fig. 6:**
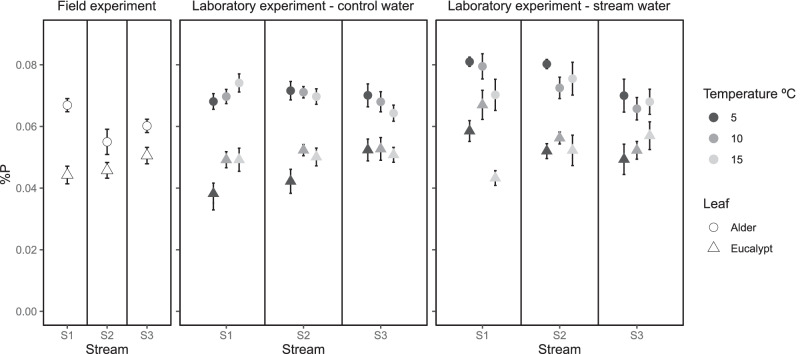
Phosphorus concentration (% DM) in alder (circle) and eucalypt (triangle) leaf discs after incubation in the field and in the laboratory (stream water vs control water at 5 °C, 10 °C and 15 °C. Note that the colour legend does not apply to field data. S1, coldest stream; S2, intermediate stream; S3, warmest stream. Mean and SE are shown

In the field experiment, %N was significantly lower for eucalypt (1.55%, mean) than alder (3.51%, mean), and %N was not related to stream water temperature (H1) (Fig. 5 and Fig. S6; Table 2). Similarly, %P was also lower for eucalypt (0.047%, mean) than alder (0.061%, mean) (Fig. 6 and Fig. S7; Table 2); however, %P showed different responses to stream water temperature depending on litter species (i.e. temperature stimulation was larger on eucalypt) (H1 and H2) (Fig. 6; Table 2). In the laboratory, %N and %P significantly differed between litter species again (Table 3): 3.42%N and 0.071%P for alder and 1.47%N and 0.050%P for eucalypt on average (Figs. 5, 6, S6 and S7). In general, water temperature did not influence %N and %P (H1). Litter discs incubated with control water had higher %P than that incubated in stream water, but it did not influence the response to temperature (H4) (Figs. 5 and 6, Figs. S6 and S7; Table 3).

## Discussion

### Temperature Effects Clearer in the Laboratory

Results from this study support previous findings on the essential role of temperature and litter quality as the main drivers of microbial decomposition in freshwater ecosystems [32, 33, 58], although some unexpected interactions appeared in our study. For instance, the increase in water temperature had a strong effect on decomposition, respiration and fungal biomass in the laboratory, but it only affected fungal biomass in the field. Moreover, decomposition of poor-quality litter (eucalypt) in the field was the highest in the stream with intermediate temperature (S2), which is difficult to associate to temperature, as this pattern was not observed in the laboratory. Fungal biomass mimicked the pattern observed for decomposition of eucalypt leaf litter in the field, with the largest fungal biomass generated in the stream with the intermediate temperature. Although fungal biomass showed the same hump shaped response for alder, this litter’s decomposition increased steadily with temperature of the stream water. It seems that the microbial community on decomposing eucalypt litter in the S3 stream seems more sensitive to low = quality substrates, since the same hump shaped relationship was observed for this microbial community in eucalypt litter in the laboratory. Although fungal biomass has been shown to be the most relevant factor explaining decomposition rates, different combinations of fungal species in an assemblage could also play a role [59]. Different effects of temperature on the structure of the fungal community depending on the substrate used (alder vs oak) have been observed previously [60]. More specifically, incubation of the same two substrates (alder and eucalypt) in three other streams in the region showed that the structure of the fungal assemblage responded to the leaf litter quality and nutrient content of the water, with some species preferring alder over eucalypt (e.g. *Flagellospora curvula*) and vice versa (e.g. *Lunulospora curvula*) [61]. These disparities of substrate preference among fungal species and differences in taxonomy and/or abundance might be behind the differential pattern for alder and eucalypt decomposition (further explored below).

### Interaction Between Temperature and Substrate Quality Clearer in the Field

Our data showed that the quality of the substrate interacted with temperature in the field experiment, as the relationship of decomposition rate with temperature was modulated by the substrate used (i.e. changed from alder to eucalypt). Contrary to our hypothesis, eucalyptus decomposition was less altered by temperature than that of alder. This result contradicts the relative consensus on increasing temperature sensitivity with decreasing litter quality (more structurally complex C substrates) based on the rationale that microbial enzymatic reactions require higher net activation energy to metabolise recalcitrant compounds [28, 29, 62]. Nevertheless, there are studies with contradicting results about the relevance of litter quality controlling temperature sensitivity in fresh waters [see 30, 33, 58, as an example]. Follstad Shah et al. [32], in a synthesis of the temperature sensitivity of litter decomposition in lotic ecosystems at the global scale, reported weak evidence of increasing temperature sensitivity with decreasing litter quality and suggested that stream attributes (e.g. availability of water, leaching of secondary toxic compounds) could eliminate or mitigate terrestrial ecosystem constraints. Some freshwater studies have reported higher leaching of secondary compounds at higher water temperature [31, 63], which would improve the palatability of poor resources at higher temperatures and enhance the positive relationship with temperature. In our study, the increase in water temperature slightly increased mass loss in the first 8 days of the conditioning in the field (22% was lost in the coldest stream and nearly 26% in the warmest). The concentration of many soluble substances, as nutrients, as well as secondary compounds that inhibit microbial growth (e.g. polyphenols, some of which have antimicrobial activity [64]) can be reduced with increasing temperatures, can modulate the growth of microbial communities. Nevertheless, we could not link fungal biomass and respiration to leaching. Thus, none of the measured parameters clearly explain why eucalypt decomposition was inhibited in the stream with the highest temperature. This local effect was also apparent for respiration and fungal biomass on alder leaf litter, but was not translated into a slower decomposition, which was the highest in the warmest stream. These results for alder suggest that structural changes can be decoupled from functional implications when facing temperature changes.

As we discussed above, water temperature influenced microbial performance (i.e. decomposition) on both types of substrates, although it was more clearly observed under laboratory conditions. Stimulation of fungal development by elevated temperatures, as we observed here, has been widely documented in studies in freshwater ecosystems [25, 65, 66]. More intriguing was the negative correlation observed between microbial respiration and water temperature; microbial assemblages from the laboratory incubations at 5 °C showed larger respiration rates at 10 °C than those incubated at 15 °C under the same conditions. Although this last finding might seem contradictory to a logical prediction of increased microbial metabolism with rising temperature [5, 24, 26], note that oxygen consumption was measured at standard temperature of 10 °C. The most plausible explanation is that the response we observed was most probably a short-term acclimation to the relative increase or decrease in temperature that measuring metabolism at standard temperature created. Some studies have described physiological “downregulation” of respiration in heterotrophic soil microbes and phytoplankton over the long-term [67, 68], suggesting that stimulatory effects of global warming on respiration rates might be lower than initially predicted as initial change rate declines over time. As we have not measured metabolism in situ in the field or laboratory at the temperatures the litters would experience in the streams, our data cannot be used to estimate the long-term response of detritus-associated microbial metabolism to temperature.

### Microbial Community Drives Decomposition But Not Sensitivity to Temperature

We expected microbial activity and decomposition rate, as well as its sensitivity to temperature, to be partially dependent on the adaptations of microbial assemblages to local environmental conditions [26, 31, 36, 40, 60]. These adaptations can come from variations of enzymatic capacities that assemblages of different taxa can have [69, 70]. Thus, stream thermal regime can become an important factor that conditions microbial activity and community structure, as species have thermal optima and specific tolerance limits for their physiological processes beyond which their activities are reduced or suppressed [26]. In our laboratory study, the overall decomposition rate differed with microbial communities coming from different streams. Nevertheless, the microbial community of origin did not interact with temperature when shaping the response of leaf decomposition and other variables. Thus, our study supports previous works reporting no or little effect of the thermal history on the sensitivity to temperature (i.e. Ea) [71, 72]. Microbial communities from temperate streams are adapted to seasonal variations in water temperature. On the other hand, thermal tolerance ranges of the microbial assemblages from streams differing in thermal regime is within the experimental thermal range (5–15 ºC). Lastly, microbial assemblages differing in specific composition can present similar leaf litter decomposition efficiencies [e.g. 61], which suggests that microbial taxonomic properties are not a determinant for the performance they present and their response to temperature.

### Water Quality Does Not Explain Decomposition Rates But Shapes Microbial Growth

Finally, our laboratory experiment also attempted to isolate the effect of water characteristics, mainly nutrient concentration, on the sensitivity of microbial activity and decomposition to temperature. The concentrations of nutrients in our streams fell within the oligotrophic-to-mesotrophic range, where nutrients stimulate decomposition [73]. Within this range, higher nutrient availability in the water can act jointly with temperature rise and result in stronger positive effects on functional processes [24, 25] and overall ecosystem functioning [74], which would imply stronger repercussions of global change on nutrient-rich streams. Our study revealed a weak role of water nutrients in mediating microbial decomposer activity and decomposition rate. In the laboratory experiment, water quality was a significant explanatory variable only for phosphorus content of the materials and fungal biomass. More surprisingly, fungal biomass in the field responded negatively to SRP and DIN:SRP. Although the change in dissolved nitrogen across the study streams (284–632 µg N L^−1^) seems large enough to stimulate the response of microbial activity and decomposition to temperature [40], the low levels of phosphorus in the waters (< 4.16 µg P L^−1^ for all streams) probably limited stronger effects of the water quality.

## Conclusions

In summary, our results emphasise the importance of temperature and leaf litter quality as drivers of microbial decomposition in fresh waters. Leaf litter quality, microbial communities and the properties of the water were able to modulate decomposition rates, but only leaf litter quality modified the sensitivity of decomposition to temperature. Our results suggest that the acceleration in microbial driven litter decomposition by global warming will be shaped by local factors, mainly by leaf litter quality, in headwater streams. However, direct extrapolation of results from laboratory to field must be careful; it translates into different patterns sometimes;, therefore, (1) more field works are needed and (2) the combination of both laboratory and field studies can provide different but valuable complementary data.

## Supplementary Information

Below is the link to the electronic supplementary material.

Supplementary file1 (DOCX 671 KB)

## Data Availability

The data will be made available on demand.

## Code Availability

It will be made available on demand.

## References

[1] IPCC (2014) Climate change 2014: synthesis report. Contribution of Working Groups I, II and III to the Fifth Assessment Report of the Intergovernmental Panel on Climate Change. IPCC, Geneva, Switzerland, Cambridge, UK

[2] Hawkins E, Ortega P, Suckling E (2017). Estimating changes in global temperature since the pre-industrial period. Bull Am Meteorol Soc.

[3] Kaushal SS, Likens GE, Jaworski NA (2010). Rising stream and river temperatures in the United States. Front Ecol Environ.

[4] Molinero J, Larrañaga A, Pérez J (2016). Stream temperature in the Basque Mountains during winter: thermal regimes and sensitivity to air warming. Clim Change.

[5] Brown JH, Gillooly JF, Allen AP (2004). Toward a metabolic theory of ecology. Ecology.

[6] Gillooly JF, Brown JH, West GB (2001). Effects of size and temperature on metabolic rate. Science (80-).

[7] Kazanjian G, Velthuis M, Aben R (2018). Impacts of warming on top-down and bottom-up controls of periphyton production. Sci Rep.

[8] Tiegs SD, Costello DM, Isken MW (2019). Global patterns and drivers of ecosystem functioning in rivers and riparian zones. Sci Adv.

[9] Yvon-Durocher G, Caffrey JM, Cescatti A (2012). Reconciling the temperature dependence of respiration across timescales and ecosystem types. Nature.

[10] Gessner MO, Swan CM, Dang CK (2010). Diversity meets decomposition. Trends Ecol Evol.

[11] Raymond PA, Zappa CJ, Butman D (2012). Scaling the gas transfer velocity and hydraulic geometry in streams and small rivers. Limnol Oceanogr Fluids Environ.

[12] Boyero L, Pearson RG, Gessner MO (2011). A global experiment suggests climate warming will not accelerate litter decomposition in streams but might reduce carbon sequestration. Ecol Lett.

[13] Boyero L, Pearson RG, Hui C (2016). Biotic and abiotic variables influencing plant litter breakdown in streams: a global study. Proc R Soc Biol Sci.

[14] Woodward G, Perkins DM, Brown LE (2010). Climate change and freshwater ecosystems: impacts across multiple levels of organization. Philos Trans R Soc Lond B Biol Sci.

[15] Wallace JB, Eggert SL, Meyer JL, Webster JR (1997). Multiple trophic levels of a forest stream linked to terrestrial litter inputs. Science (80- ).

[16] Perkins DM, Reiss J, Yvon-Durocher G, Woodward G (2010). Global change and food webs in running waters. Hydrobiologia.

[17] Tank JL, Rosi-Marshall EJ, Griffiths NA (2010). A review of allochthonous organic matter dynamics and metabolism in streams. J North Am Benthol Soc.

[18] Pascoal C, Cássio F (2004). Contribution of fungi and bacteria to leaf litter decomposition in a polluted river. Appl Environ Microbiol.

[19] Tank CJ, Rosemond AD, Mehring AS (2015). The role of aquatic fungi in transformations of organic matter mediated by nutrients. Freshw Biol.

[20] Gessner MO, Gulis V, Kuehn KA, Kubicek CP, Druzhinin IS (2007). Fungal decomposers of plant litter in aquatic ecosystems. The Mycota: Environmental and Microbial Relationships.

[21] Tiegs SD, Costello DM, Isken MW (2019). Global patterns and drivers of ecosystem functioning in rivers and riparian zones. Sci Adv.

[22] Amani M, Graça MAS, Fereira V (2019). Effects of elevated atmospheric CO 2 concentration and temperature on litter decomposition in streams: a meta-analysis. Int Rev Hydrobiol.

[23] Fernandes I, Uzun B, Pascoal C, Cássio F (2009). Responses of aquatic fungal communities on leaf litter to temperature-change events. Int Rev Hydrobiol.

[24] Manning DWP, Rosemond AD, Gulis V (2018). Nutrients and temperature additively increase stream microbial respiration. Glob Chang Biol.

[25] Ferreira V, Chauvet E (2011). Synergistic effects of water temperature and dissolved nutrients on litter decomposition and associated fungi. Glob Chang Biol.

[26] Canhoto C, Gonçalves AL, Bärlocher F (2016). Biology and ecological functions of aquatic hyphomycetes in a warming climate. Fungal Ecol.

[27] Shah JJF, Swan CM, Boyero L, Canhoto C (2021). Individual and interacting effects of elevated CO2, warming, and hydrologic intensification on leaf litter decomposition in streams. The Ecology of Plant Litter Decomposition in Stream Ecosystems.

[28] Fierer N, M CJ, McLauchlan J, Schimel JP (2005) Litter quality and the temperature sensitivity of decomposition. Ecology 86:320–326. 10.1890/04-1254

[29] Conant RT, Ryan MG, Birge HE (2011). Temperature and soil organic matter decomposition rates – synthesis of current knowledge and a way forward. Glob Chang Biol.

[30] Wetterstedt JA, Peterson T, Agren GI (2010). Temperature sensitivity and substrate quality in soil organic matter decomposition: results of an incubation study with three substrates. Glob Chang Biol.

[31] Fernandes I, Pascoal C, Guimaraes H (2012). Higher temperature reduces the effects of litter quality on decomposition by aquatic fungi. Freshw Biol.

[32] Follstad Shah JJ, Kominoski JS, Ardón M (2017). Global synthesis of the temperature sensitivity of leaf litter breakdown in streams and rivers. Glob Chang Biol.

[33] Fenoy E, Casas JJ, Díaz-López M (2016). Temperature and substrate chemistry as major drivers of interregional variability of leaf microbial decomposition and cellulolytic activity in headwater. FEMS Microbiol Ecol.

[34] Ferreira V, Chauvet E (2011). Future increase in temperature more than decrease in litter quality can affect microbial litter decomposition in streams. Oecologia.

[35] Martínez A, Monroy S, Pérez J (2016). In-stream litter decomposition along an altitudinal gradient: does substrate quality matter?. Hydrobiologia.

[36] Dang CK, Schindler M, Chauvet E, Gessner MO (2009). Temperature oscillation coupled with fungal community shifts can modulate warming effects on litter decomposition. Ecology.

[37] Geraldes P, Pascoal C, Cássio F (2012). Effects of increased temperature and aquatic fungal diversity on litter decomposition. Fungal Ecol.

[38] Bradford MA (2013). Thermal adaptation of decomposer communities in warming soils. Front Microbiol.

[39] Strickland MS, Keiser AD, Bradford MA (2015). Climate history shapes contemporary leaf litter decomposition. Biogeochemistry.

[40] Martínez A, Larrañaga A, Pérez J (2014). Temperature affects leaf litter decomposition in low-order forest streams: field and microcosm approaches. FEMS Microbiol Ecol.

[41] Crowther TW, Maynard DS, Crowther TR (2014). Untangling the fungal niche: the trait-based approach. Front Microbiol.

[42] APHA (American Public Health Association) (2005) Standard methods for the examination of water and wastewater, 21st ed. American Public Health Association, American Water Works Association, and Water Environment Federation, Washington, D.C

[43] Graça MAS, Pozo J, Canhoto C, Elosegi A (2002). Effects of Eucalyptus plantations on detritus, decomposers, and detritivores in streams. Sci World J.

[44] Pozo J, Basaguren A, Elosegui A (1998). Afforestation with Eucalyptus globulus and leaf litter decomposition in streams of northern Spain. Hydrobiologia.

[45] Pozo J, Gonzalez E, Díez J (1997). Inputs of particulate organic matter to streams with different riparian vegetation. J North Am Benthol Soc.

[46] Ferreira V, Boyero L, Calvo C (2019). A global assessment of the effects of Eucalyptus plantations on stream ecosystem functioning. Ecosystems.

[47] Abelho M (2001). From litterfall to breakdown in streams: a review. Sci World J.

[48] Allen SE, Grimshaw HM, Parkinson JA, Quarmby C (1974). Chemical analysis of ecological materials.

[49] Gessner MO (2020) Ergosterol as measure of fungal biomass. In: Baerlocher F, Gessner MO, Graça MAS (eds) Methods to study litter decomposition, 2st ed. Springer International Publishing, pp 247–245

[50] Gessner MO, Chauvet E (1993). Ergosterol-to-biomass conversion factors for aquatic hyphomycetes. Appl Environ Microbiol.

[51] Zar JH (2010). Biostatistical analysis.

[52] Baerlocher F (2020) Leaf mass loss estimated by the litter bag technique. In: Baerlocher F, Gessner MO, Graça MAS (eds) Methods to study litter decomposition, 2sd ed. Springer International Publishing, pp 43–51

[53] Bates D, Maechler M, Bolker B, Walker S (2015). Fitting linear mixed-effects models using lme4. J Stat Softw.

[54] Zuur AF, Ieno EN, Walker NJ (2009). Mixed effects models and extensions in ecology with R.

[55] Luke SG (2017) Evaluating significance in linear mixed-effects models in R. Behav Res Methods 49:. 10.3758/s13428-016-0809-y 10.3758/s13428-016-0809-y 27620283

[56] Pinheiro JC, Bates DM (2000). Linear mixed-effects models: basic concepts and examples. Mixed-effects models in S and S-Plus.

[57] R Core Team (2015) R: A Language and environmental for statistical computing

[58] García-Palacios P, McKie BG, Handa IT (2016). The importance of litter traits and decomposers for litter decomposition: a comparison of aquatic and terrestrial ecosystems within and across biomes. Funct Ecol.

[59] Duarte S, Pascoal C, Cássio F, Bärlocher F (2006). Aquatic hyphomycete diversity and identity affect leaf litter decomposition in microcosms. Oecologia.

[60] Gonçalves AL, Graça MAS, Canhoto C (2013). The effect of temperature on leaf decomposition and diversity of associated aquatic hyphomycetes depends on the substrate. Fungal Ecol.

[61] Chauvet E, Fabre E, Elósegi A, Pozo J (1997). The impact of eucalypt on the leaf-associated aquatic hyphomycetes in Spanish streams. Can J Bot.

[62] Conant RT, Drijber RA, Haddix ML (2008). Sensitivity of organic matter decomposition to warming varies with its quality. Glob Chang Biol.

[63] Mas-Martí E, Muñoz I, Oliva F, Canhoto C (2015). Effects of increased water temperature on leaf litter quality and detritivore performance : a whole-reach manipulative experiment. Freshw Biol.

[64] Canhoto C, Graça MAS (1999). Leaf barriers to fungal colonization and shredders (Tipula lateralis) consumption of decomposing Eucalyptus globulus. Microb Ecol.

[65] Fernandes I, Seena S, Pascoal C, Cássio F (2014). Elevated temperature may intensify the positive effects of nutrients on microbial decomposition in streams. Freshw Biol.

[66] Ferreira V, Chauvet E, Canhoto C (2015). Effects of experimental warming, litter species, and presence of macroinvertebrates on litter decomposition and associated decomposers in a temperate mountain stream. Can J Fish Aquat Sci.

[67] Padfield D, Yvon-Durocher G, Buckling A (2016). Rapid evolution of metabolic traits explains thermal adaptation in phytoplankton. Ecol Lett.

[68] Crowther TW, Bradford MA (2013). Thermal acclimation in widespread heterotrophic soil microbes. Ecol Lett.

[69] Chamier AC (1985) Cell-wall-degrading enzymes of aquatic hyphomycetes: a review. Bot J Linn Soc 91. 10.1111/j.1095-8339.1985.tb01136.x

[70] Suberkropp K, Arsuffi TL, Anderson JP (1983) Comparison of degradative ability, enzymatic activity, and palatability of aquatic hyphomycetes grown on leaf litter. Appl Environ Microbiol 46. 10.1128/aem.46.1.237-244.1983 10.1128/aem.46.1.237-244.1983 PMC239294 16346343

[71] Sand-Jensen K, Pedersen NL, Søndergaard M (2007). Bacterial metabolism in small temperate streams under contemporary and future climates. Freshw Biol.

[72] Perkins DM, Yvon-Durocher G, Demars BOL (2012). Consistent temperature dependence of respiration across ecosystems contrasting in thermal history. Glob Chang Biol.

[73] Gulis V, Ferreira V, Graça MAS (2006) Stimulation of leaf litter decomposition and associated fungi and invertebrates by moderate eutrophication: implications for stream assessment. Freshw Biol 1655–1669. 10.1111/j.1365-2427.2006.01615.x

[74] Cross WF, Hood JM, Benstead JP (2015). Interactions between temperature and nutrients across levels of ecological organization. Glob Chang Biol.

