# Supplementary material for: Temperature Sensitivity of Microbial Litter Decomposition in Freshwaters: Role of Leaf Litter Quality and Environmental Characteristics
Source: Microb Ecol. 2022 Jun 2;85(3):839–52. doi: 10.1007/s00248-022-02041-5 (PMC10156624; doi:10.1007/s00248-022-02041-5)

**SUPPORTING INFORMATION**

**Temperature sensitivity of microbial litter decomposition in freshwaters: role of leaf litter quality and environmental characteristics**

Silvia Monroy^1*^, Aitor Larrañaga^1^, Aingeru Martínez^1^, Javier Pérez ^1^, Jon Molinero ^2^, Ana Basaguren^1^ and Jesús Pozo^1^

Affiliations:

^1^Department of Plant Biology and Ecology, Faculty of Science and Technology, University of the Basque Country, P.O. Box 644, 48080 Bilbao, Spain

^2^Escuela de Gestión Ambiental, Pontificia Universidad Católica del Ecuador Sede Esmeraldas, 080150 Esmeraldas, Ecuador

*Corresponding author: Silvia Monroy; silvia.monroy@ehu.eus; ORCID: 0000-0002-7430-1776

**Table S1.** Results of linear mixed models performed on ln-transformed leaf decomposition of alder and eucalypt leaf discs incubated in the field and the laboratory microcosms at three temperatures (5, 10 and 15°C). Multicollinearity was first removed with VIF scores and models were then simplified and non-significant interactions removed, except for temperature, which was kept in all models. Significant values (p < 0.05) are highlighted in bold. InvTemp = temperature expressed in terms of metabolic theory of ecology [3]. Temperature was centered at 10°C [1/kTc -1/kT, where Tc and T is the normalization and observed temperature in Kelvin, respectively, and multiplied by the Boltzmann constant ,k=8.62 × 10^-5^ eV K^-1^].

|  |  | **Df** | **F value** | **p-value** | **Coef. sign** | **Interpretation** |
| --- | --- | --- | --- | --- | --- | --- |
| Field decomposition (Ln) |  |  |  |  |  |  |
|  | InvTemp | 1,62 | 3.44 | 0.0684 |  |  |
|  | Leaf | 1,62 | 48.29 | **< 0.0001** | **-** | Alder > Euc |
|  | DIN | 1,62 | 6.88 | **0.0110** | **-** | DIN inhibits |
|  | Leaf:DIN | 1,62 | 11.15 | 0.0014 | - | DIN inhibits more eucalypt |
| Lab decomposition (Ln) |  |  |  |  |  |  |
|  | InvTemp | 1,224 | 49.71 | **< 0.0001** | **-** | Temperature stimulates |
|  | Leaf | 1,224 | 74.59 | **< 0.0001** | **-** | Alder > Euc |

**Table S2.** K rates and Ea with 95% confidence intervals (CI) for all treatments.

| **Approach** | **Water** | **Leaf** | **Stream** | **Temp.** | **k rates** | | **Ea** | | |  |
| --- | --- | --- | --- | --- | --- | --- | --- | --- | --- | --- |
|  |  |  |  | (^o^C) | Mean | CI | | Mean | CI | |
| Field |  | Alder | S1 |  | 0.00677 | 0.00280 | | 0.7185 | 0.553 | |
|  |  |  | S2 |  | 0.00722 | 0.00106 | |  |  | |
|  |  |  | S3 |  | 0.00938 | 0.00168 | |  |  | |
|  |  | Eucalypt | S1 |  | 0.00334 | 0.00130 | | -0.3081 | 0.818 | |
|  |  |  | S2 |  | 0.00426 | 0.00093 | |  |  | |
|  |  |  | S3 |  | 0.00232 | 0.00087 | |  |  | |
|  |  |  |  |  |  |  | |  |  | |
| Laboratory | Control | Alder | S1 | 5 | 0.00250 | 0.00056 | | 1.065 | 0.812 | |
|  |  |  |  | 10 | 0.00480 | 0.00281 | |  |  | |
|  |  |  |  | 15 | 0.00483 | 0.00122 | |  |  | |
|  |  |  | S2 | 5 | 0.00211 | 0.00048 | | 1.338 | 0.72 | |
|  |  |  |  | 10 | 0.00355 | 0.00086 | |  |  | |
|  |  |  |  | 15 | 0.00561 | 0.00142 | |  |  | |
|  |  |  | S3 | 5 | 0.00250 | 0.00075 | | 0.8614 | 1.387 | |
|  |  |  |  | 10 | 0.00318 | 0.00092 | |  |  | |
|  |  |  |  | 15 | 0.00547 | 0.00186 | |  |  | |
|  |  | Eucalypt | S1 | 5 | 0.00114 | 0.00044 | | 0.4183 | 0.472 | |
|  |  |  |  | 10 | 0.00351 | 0.00217 | |  |  | |
|  |  |  |  | 15 | 0.00184 | 0.00046 | |  |  | |
|  |  |  | S2 | 5 | 0.00204 | 0.00090 | | 0.5458 | 0.478 | |
|  |  |  |  | 10 | 0.00377 | 0.00296 | |  |  | |
|  |  |  |  | 15 | 0.00492 | 0.00273 | |  |  | |
|  |  |  | S3 | 5 | 0.00091 | 0.00075 | | 3.452 | 1 | |
|  |  |  |  | 10 | 0.00133 | 0.00049 | |  |  | |
|  |  |  |  | 15 | 0.00161 | 0.00040 | |  |  | |
|  | Stream | Alder | S1 | 5 | 0.00345 | 0.00078 | | 0.3216 | 0.158 | |
|  |  |  |  | 10 | 0.00411 | 0.00117 | |  |  | |
|  |  |  |  | 15 | 0.00548 | 0.00081 | |  |  | |
|  |  |  | S2 | 5 | 0.00190 | 0.00079 | | 0.5085 | 0.677 | |
|  |  |  |  | 10 | 0.00260 | 0.00140 | |  |  | |
|  |  |  |  | 15 | 0.00526 | 0.00586 | |  |  | |
|  |  |  | S3 | 5 | 0.00326 | 0.00020 | | 0.5009 | 0.196 | |
|  |  |  |  | 10 | 0.00420 | 0.00146 | |  |  | |
|  |  |  |  | 15 | 0.00684 | 0.00219 | |  |  | |
|  |  | Eucalypt | S1 | 5 | 0.00155 | 0.00221 | | 0.4901 | 0.564 | |
|  |  |  |  | 10 | 0.00191 | 0.00113 | |  |  | |
|  |  |  |  | 15 | 0.00240 | 0.00058 | |  |  | |
|  |  |  | S2 | 5 | 0.00198 | 0.00215 | | 0.5402 | 1.054 | |
|  |  |  |  | 10 | 0.00198 | 0.00097 | |  |  | |
|  |  |  |  | 15 | 0.00237 | 0.00022 | |  |  | |
|  |  |  | S3 | 5 | 0.00096 | 0.00055 | | 0.5523 | 0.391 | |
|  |  |  |  | 10 | 0.00187 | 0.00063 | |  |  | |
|  |  |  |  | 15 | 0.00210 | 0.00120 | |  |  | |

**Fig S1.** Simplified laboratory experimental design for each of leaf material microbially conditioned in each study stream. Alder (A) and eucalypt (E) leaf discs incubated in microcosms with control water (water from additional control stream; grey) and stream water (water from conditioning stream; white) at 5, 10 and 15°C. Twenty microcosms with control water (three samplings) and eight with stream water (one sampling).

**
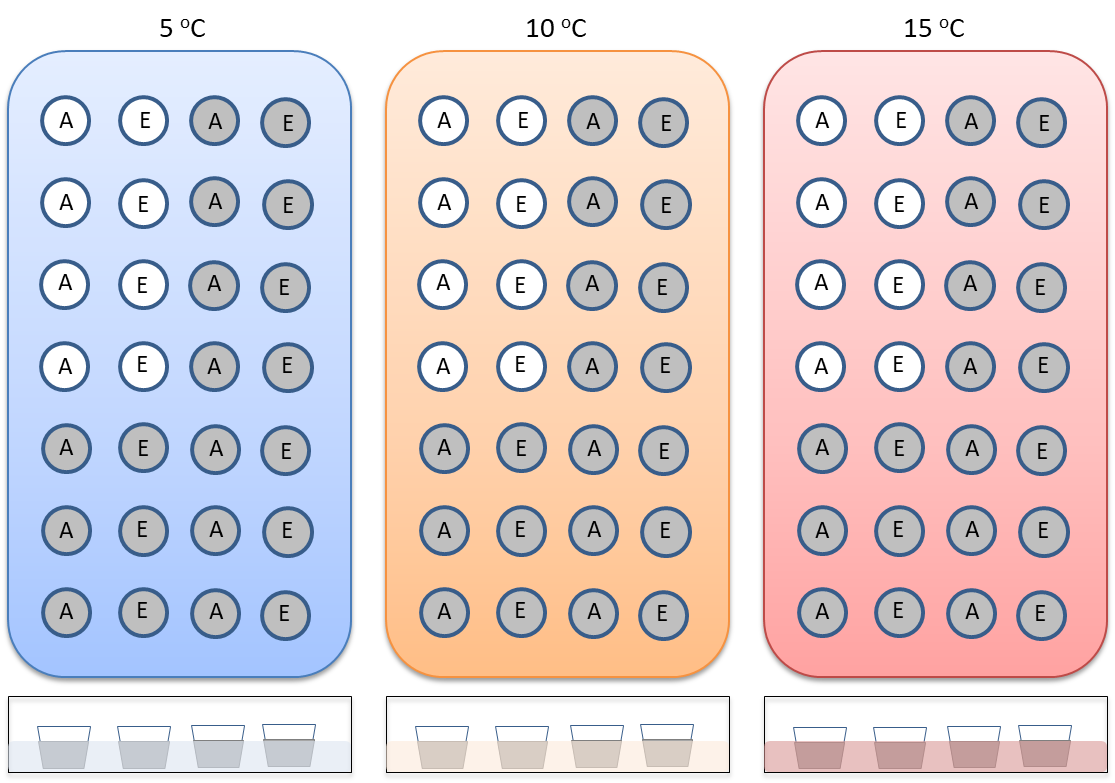
**

**Fig. S2.** AFDM remaining (%) of alder (white) and eucalypt (black) litter from incubation in the field (left) and in the laboratory (right) at 5°C (circle), 10°C (square) and 15°C (triangle) on each sampling date. In the laboratory experiment, the points to the left of the dashed line show the AFDM remaining dynamics in leaf discs incubated in control water (CW), and the right ones % AFDM remaining of those incubated in stream water (SW). S1, coldest stream; S2, intermediate stream; S3, warmest stream. Mean ± SE. Note the different scale.


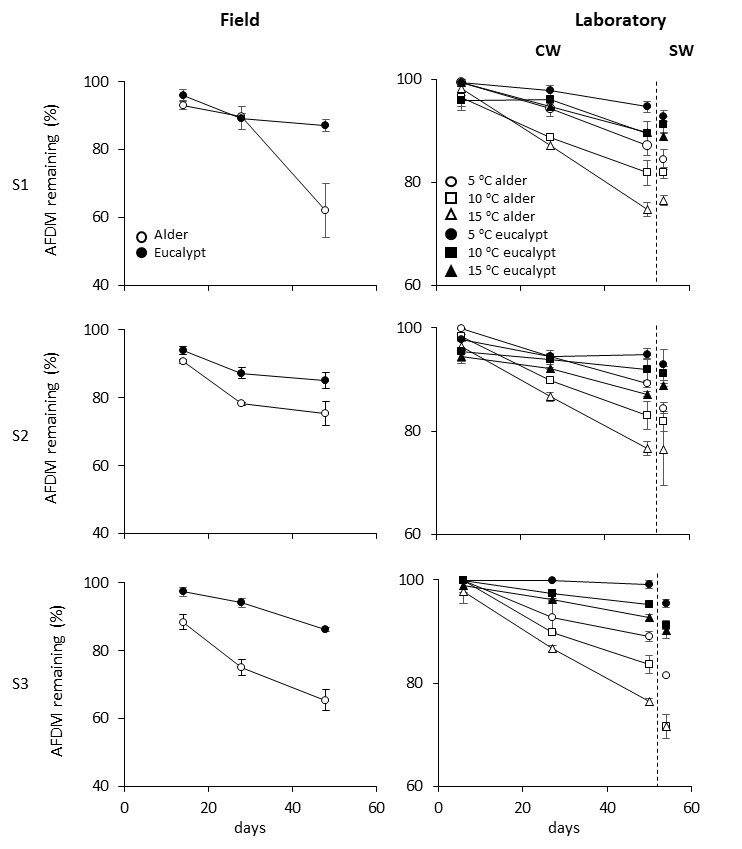


**Fig. S3.** Relationships between the natural logarithm of leaf decomposition rate and water temperature expressed as the inverse absolute temperature in Kelvin (T) multiplied by Boltzmann constant (k, 8.62 × 10^-5^ eV K^-1^). Data from field experiment (a) for alder (grey dots) and eucalypt litter (black dots). Data from laboratory experiment for alder (b) and eucalypt litter (c), microbially conditioned in the three streams (S1: black; S2: red; S3: blue) and incubated in stream water (dashed lines) or control water (solid lines). Significant regression lines are show.

**
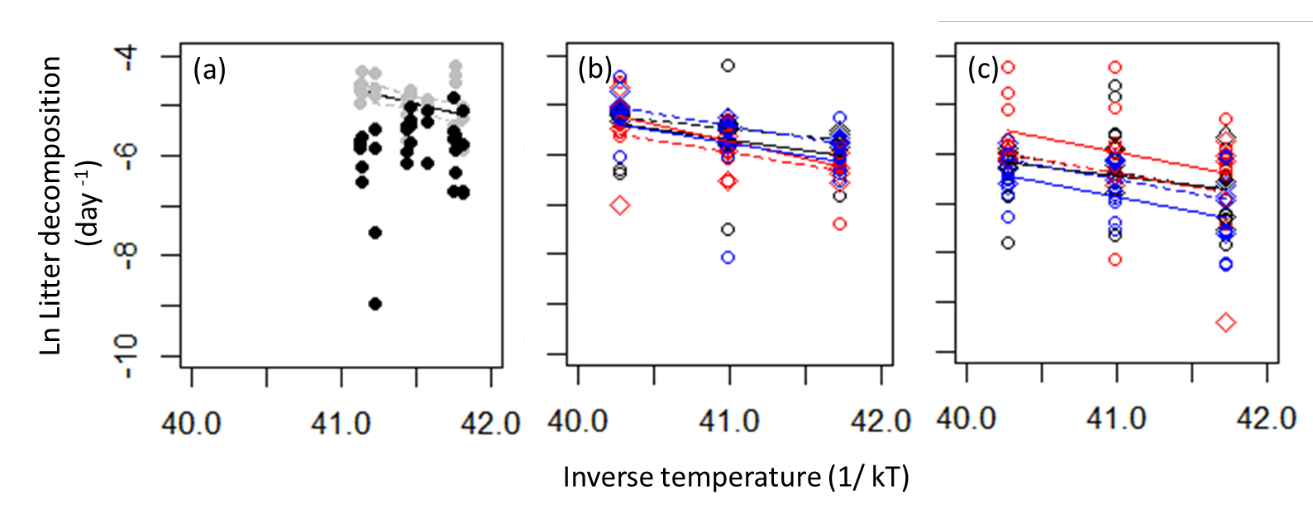
**

**Fig. S4.** Oxygen consumption (mg O_2_ g^-1^ DM h^-1^) on alder (white) and eucalypt (black) leaf discs incubated in the field (left) and in the laboratory (right) at 5°C (circle), 10°C (square) and 15°C (triangle). In the laboratory experiment, the points to the left of the dashed line show the oxygen consumption dynamic in leaf discs incubated in control water (CW) and, the right ones show the oxygen consumption in leaf discs incubated in stream water (SW) at the end of the experiment. S1, coldest stream; S2, intermediate stream; S3, warmest stream. Mean ± SE.


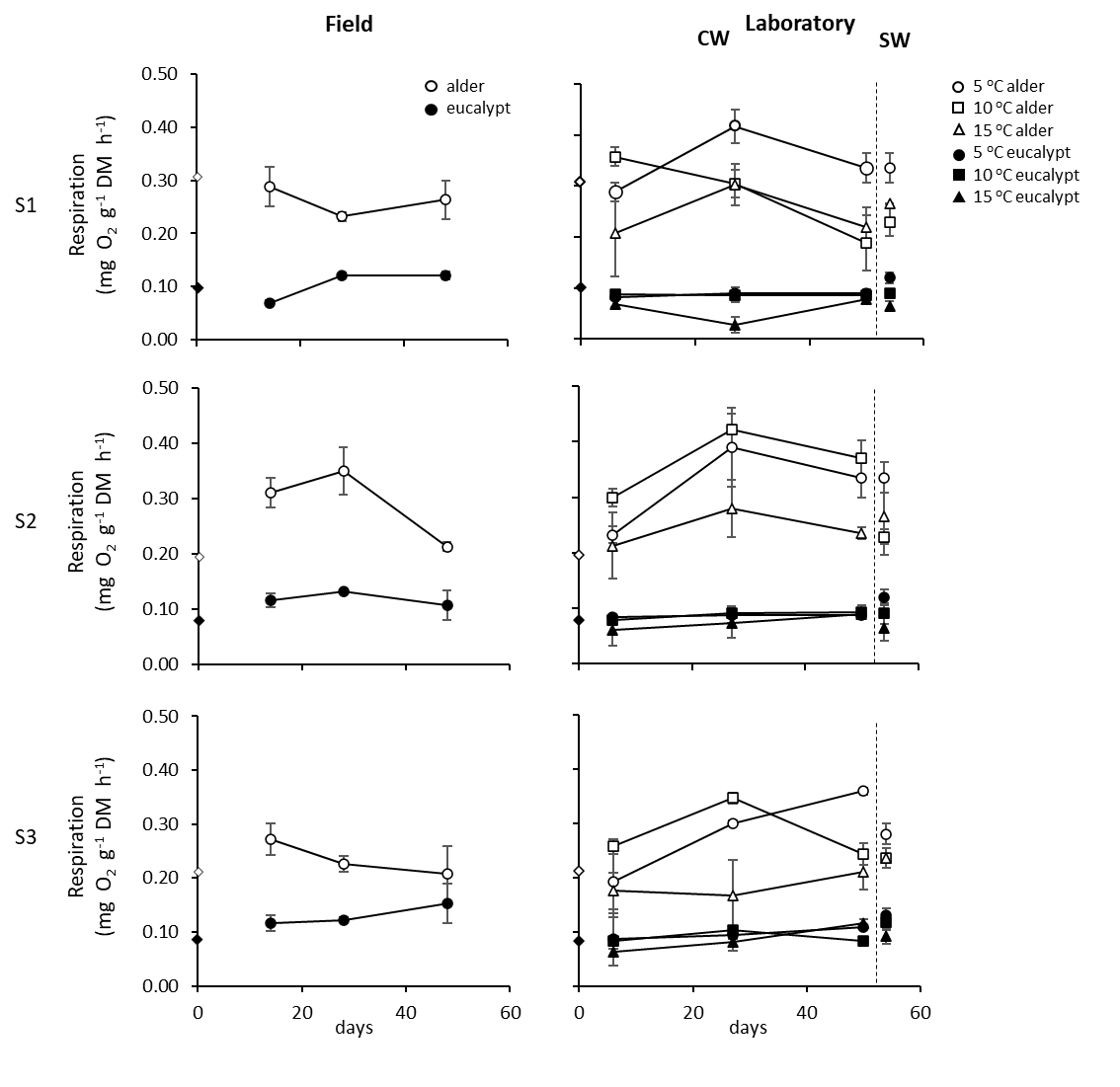


**Fig. S5.** Fungal biomass accrual (mg g^-1^ DM) on alder (white) and eucalypt (black) leaf discs incubated in the field (left) and in the laboratory (right) at 5°C (circle), 10°C (square) and 15°C (triangle). In the laboratory experiment, the points to the left of the dashed line show the fungal biomass dynamic on leaf discs incubated in control water (CW) and, the right ones show the fungal biomass on leaf discs incubated in stream water (SW) at the end of the experiment. S1, coldest stream; S2, intermediate stream; S3, warmest stream. Mean ± SE.


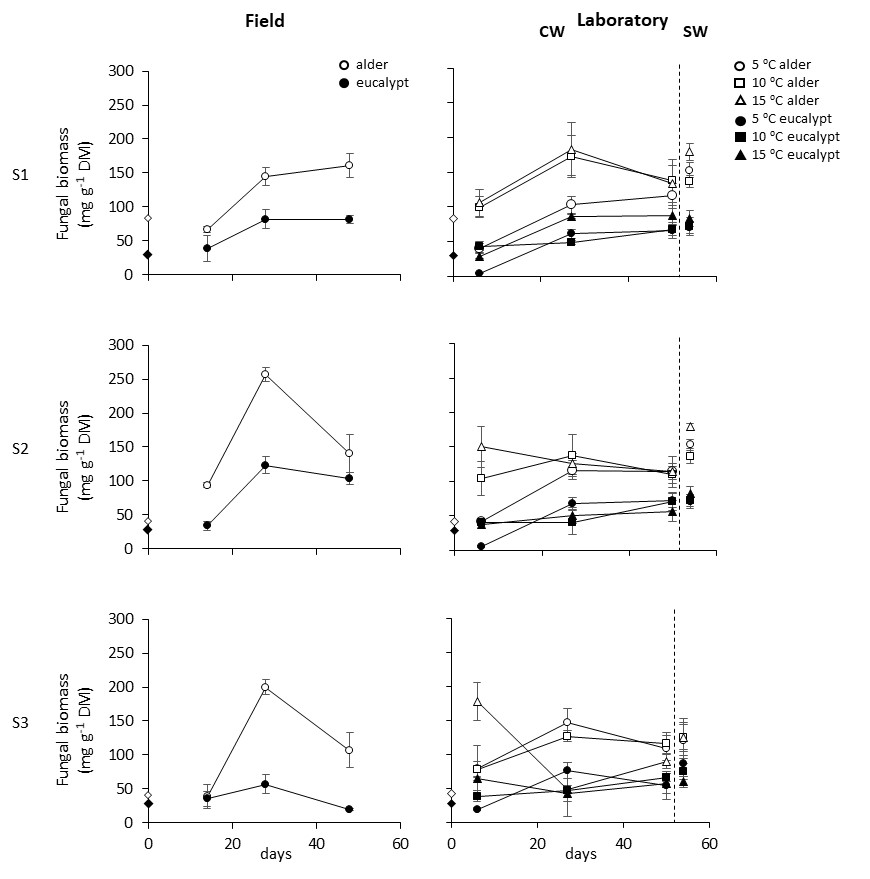


.

**Fig. S6.** Nitrogen concentration (% DM) in alder (white) and eucalypt (black) leaf discs incubated in the field (left) and in the laboratory (right) at 5°C (circle), 10°C (square) and 15°C (triangle). In the laboratory experiment, the points to the left of the dashed line show the N dynamic of leaf discs incubated in control water (CW) and, the right ones show the N of leaf discs incubated in stream water (SW) at the end of the experiment. S1, coldest stream; S2, intermediate stream; S3, warmest stream. Mean ± SE.

**
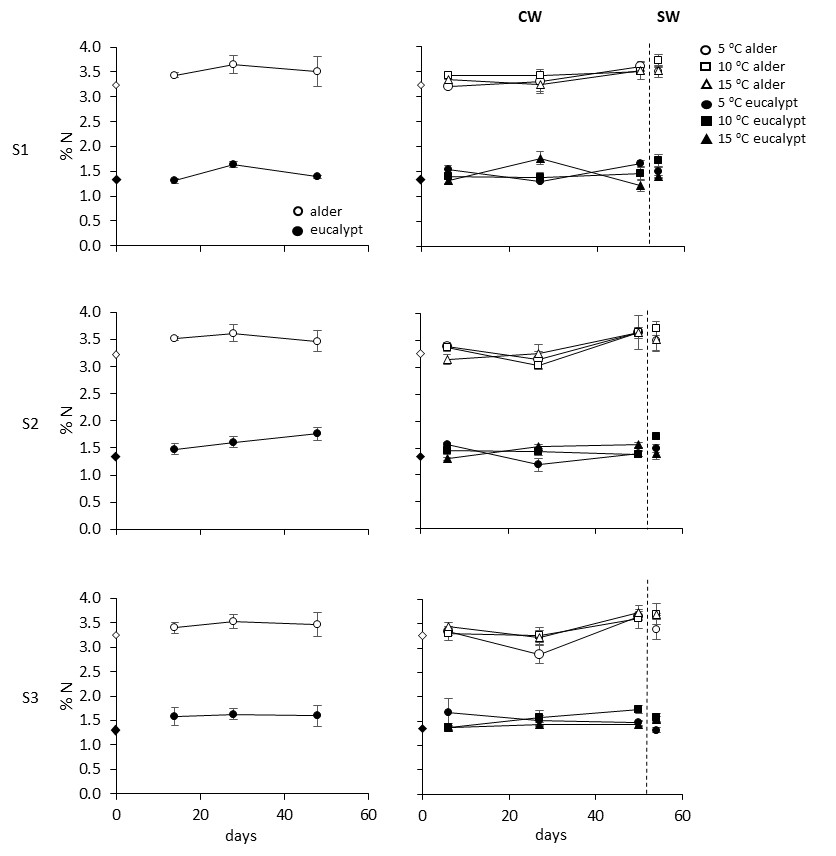
**

.

**Fig. S7.** Phosphorus concentration (% DM) in alder (white) and eucalypt (black) leaf discs incubated in the field (left) and in the laboratory (right) at 5°C (circle), 10°C (square) and 15°C (triangle). In the laboratory experiment, the points to the left of the dashed line show the P dynamic of leaf discs incubated in control water (CW) and, the right ones show the P of leaf discs incubated in stream water (SW) at the end of the experiment. S1, coldest stream; S2, intermediate stream; S3, warmest stream. Mean ± SE.


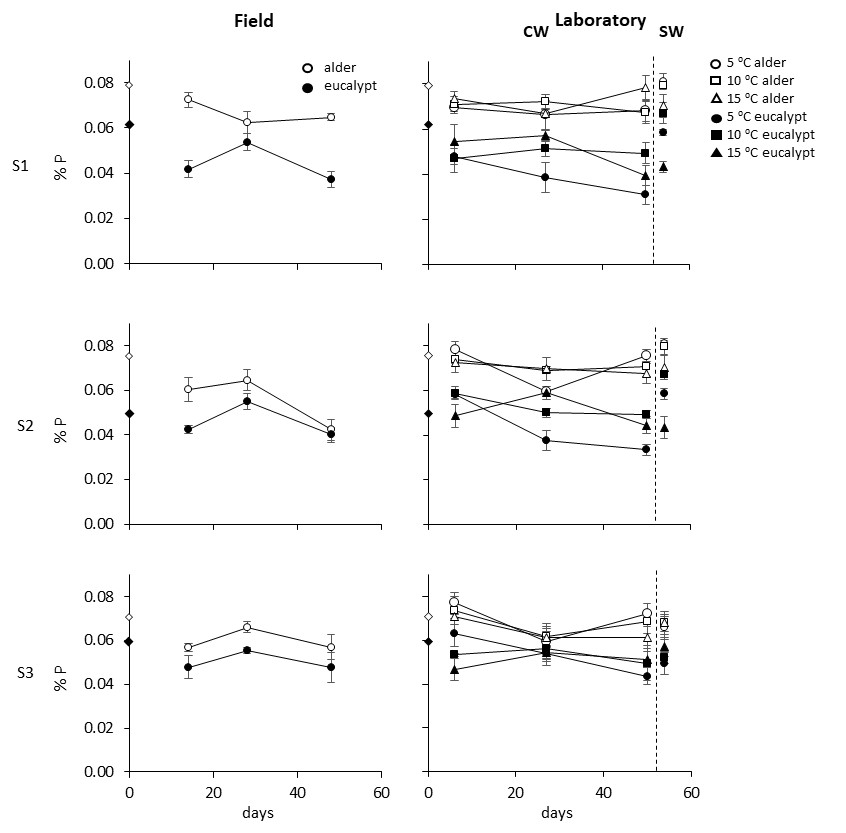

Supplement: Supplementary file 1 — Supplementary file1 (DOCX 671 KB) [file 248_2022_2041_MOESM1_ESM.docx]
